# Significance of Sex Differences in ncRNAs Expression and Function in Pregnancy and Related Complications

**DOI:** 10.3390/biomedicines9111509

**Published:** 2021-10-20

**Authors:** Rosaria Varì, Beatrice Scazzocchio, Tiziana Filardi, Anna Citarella, Maria Bellenghi, Roberta Masella, Carmela Santangelo

**Affiliations:** 1 Center for Gender-Specific Medicine, Istituto Superiore di Sanità, Viale Regina Elena 299, 00161 Rome, Italy; rosaria.vari@iss.it (R.V.); beatrice.scazzocchio@iss.it (B.S.); maria.bellenghi@iss.it (M.B.); roberta.masella@iss.it (R.M.); 2 Department of Experimental Medicine, Sapienza University, Viale del Policlinico 155, 00161 Rome, Italy; tiziana.filardi@uniroma1.it; 3 Department of Molecular Medicine, Sapienza University of Rome, Viale Regina Elena 291, 00161 Rome, Italy; anna.citarella@uniroma1.it

**Keywords:** ncRNA, miRNA, pregnancy complications, placenta, embryo, fetal sex, gestational diabetes, preeclampsia

## Abstract

In the era of personalized medicine, fetal sex-specific research is of utmost importance for comprehending the mechanisms governing pregnancy and pregnancy-related complications. In recent times, noncoding RNAs (ncRNAs) have gained increasing attention as critical players in gene regulation and disease pathogenesis, and as candidate biomarkers in human diseases as well. Different types of ncRNAs, including microRNAs (miRNAs), piwi-interacting RNAs (piRNAs), long noncoding RNAs (lncRNAs), and circular RNAs (circRNAs), participate in every step of pregnancy progression, although studies taking into consideration fetal sex as a central variable are still limited. To date, most of the available data have been obtained investigating sex-specific placental miRNA expression. Several studies revealed that miRNAs regulate the (patho)-physiological processes in a sexually dimorphic manner, ensuring normal fetal development, successful pregnancy, and susceptibility to diseases. Moreover, the observation that ncRNA profiles differ according to cells, tissues, and developmental stages of pregnancy, along with the complex interactions among different types of ncRNAs in regulating gene expression, strongly indicates that more studies are needed to understand the role of sex-specific ncRNA in pregnancy and associated disorders.

## 1. Introduction

Pregnancy is a physiological state in which numerous and complex modifications occur in the mother in order to accommodate the growing fetus [1]. According to the theory of the fetal origin of adult diseases (FOAD), hypothesized by David Barker, the intrauterine environment plays a relevant role in fetal growth and development and markedly contributes to the risk of developing diseases later in life [2]. It is well known that fetal growth and development occur in a sexually dimorphic manner [3]. However, growing evidence indicates that fetal sex differently influences the course of pregnancy and associated complications, as well maternal health [4,5,6,7]. Although ethnic differences exist in the maternal response, several data indicate that women carrying a male show an increased risk of gestational diabetes (GDM), placental abruption [7,8], and preterm birth [9], whereas gestational hypertension/preeclampsia (PE) appear to be linked to carrying a female fetus [10]. Genetics, nutrition, and other known and not yet completely known environmental factors contribute to successful pregnancy [1,11]. Deregulation of the complex interactions between these factors is responsible for the onset of pregnancy-related complications with long-term impact on offspring health until adulthood, as well as on the mother’s health [1,11]. Despite it being known that the biological differences between sexes become evident early in embryogenesis, the processes involved in these sex differences are not completely known [9]. Knowledge about the mechanisms through which female and male fetuses respond to external and internal stimuli or insults during pregnancy is rather fragmentary [12]. Embryo development requires proliferation and differentiation processes that are tightly regulated in time and duration [12,13]. Several mechanisms and numerous factors, including an increasing number of noncoding RNAs (ncRNAs), modulate gene transcription at every stage of embryo and fetus development [13] and pregnancy progression [14]. Non-coding RNAs play a significant role in epigenetic modifications and exhibit dynamic expression patterns in the regulation of biological processes [13]. They are expressed by almost all cells, allowing cell-to-cell interactions, and are detected in body fluids [15]. In recent years, ncRNAs have been studied as promising diagnostic and prognostic factors, as well as being studied as therapeutic targets in tumors [15,16] and neurodegenerative and metabolic disorders [17], thus representing an area of intensive ongoing investigation in the context of human physiology and pathophysiology. The growing evidence of the importance of sexual dimorphism in health and disease imposes a wider knowledge of the biological processes underlying sex differences.

In this review, we summarize the latest data on the involvement of ncRNAs in pregnancy and related complications according to fetal sex, with the aim of giving an overview of how this class of RNAs can influence placental function and embryonic development by regulating gene expression. In addition, the role of ncRNAs in modulating pregnancy-associated maternal disorders has also been reviewed in order to provide useful remarks for future studies.

## 2. ncRNAs as Potential Biomarkers

**Figure 1:**
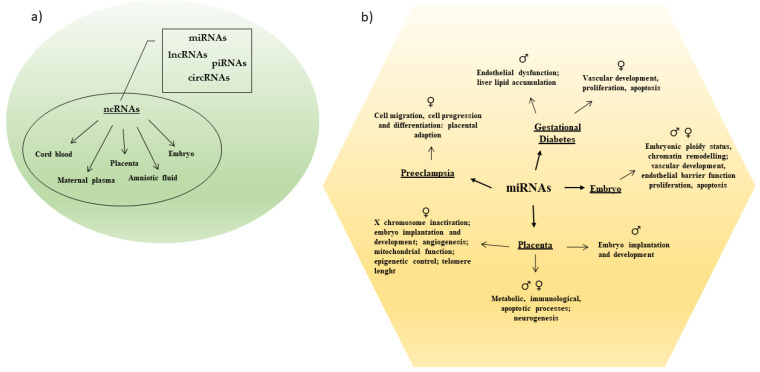
(**a**) Different categories of ncRNAs have been detected in maternal and fetal body tissues. (**b**) Sex-specific miRNAs regulate several processes in pregnancy and pregnancy related complications.

Only 1–2% of the mammalian genome encodes for proteins [17]. A larger part of the genome is transcribed into ncRNAs that actively participate in the maintenance of cellular functionality. Noncoding RNAs modulate different biological processes due their capability to regulate gene expression at both the transcriptional and post-transcriptional level [17]. They modulate cell differentiation and sex differentiation and sex-specific tissue development and/or function, as well as initiation and progression of multiple diseases [17,18] (Figure 1). 

According to the transcript length, ncRNAs are classified into (i) small ncRNAs (sncRNAs; <200 nucleotides, nt), which include microRNAs (miRNAs), small interfering RNAs (siRNAs), piwi-interacting RNAs (piRNAs), small nuclear RNAs (snRNAs), small nucleolar RNAs (snoRNAs), and vault-RNAs (VT-RNAs); and (ii) long ncRNAs (lncRNAs; ≥200 nt), including circular RNAs (circRNAs) [19,20]. All of these ncRNAs differently contribute to cellular homeostasis, influencing embryo development and cell regulation [19,20]. Aberrant expression patterns of both snc- and lncRNAs have been observed in various pathological conditions, and a significantly altered expression has been detected in biological fluids, making them possible diagnostic and prognostic biomarkers and/or therapeutic targets in human diseases [15,16,17,21]. To date, miRNAs and lncRNAs are the most studied ncRNAs because of their involvement in cancer development and progression [22], as well as in embryo and fetus development [13,19]. Both ncRNA groups exhibit tissue and temporally specific expression patterns and regulate each other through their binding sites, both in cytoplasm and nucleus, thus influencing cell-cycle progression, proliferation, apoptosis, and development [23].

MicroRNAs are single-stranded ncRNAs (~21–24 nt) that regulate gene expression at the post-transcriptional level by silencing specific target RNAs or by targeting regulatory RNAs such as lncRNAs [22]. Each miRNA can target hundreds or thousands of mRNAs, and their deregulation is associated with an increasing number of human diseases [24,25,26], including several pregnancy-associated disorders [14,27] namely, spontaneous preterm birth [28], small-for-gestational-age (SGA) births [29], ectopic pregnancy [30], GDM [31], and PE [32].

PIWI-interacting RNAs (piRNAs) are another class of short ncRNAs (26–31 nt) involved in the differentiation of embryonic stem cells and in early embryonic development. The piRNA pathway is essential for germ line survival and genome integrity due to its capability of regulating the expression of downstream target genes [33]. PIWI-piRNAs are expressed in the human fetal ovary and adult testis, and a subset of piRNAs genes are expressed in a sex-specific manner [34,35]. PIWI-piRNAs also have a key role in maternal-to-zygotic transition (MZT). In the early embryo, they contribute to the massive maternal mRNA degradation that is necessary for allowing activation of the zygote genome [36]. Of note, recent evidence indicates that piRNAs are also expressed in human somatic cells in a tissue-specific manner and are involved in the development of cardiomyopathies, cancer, infectious diseases [37], and neurodegenerative diseases since they participate in the maintenance of metabolic homeostasis and in neurodevelopment [38]. The presence of piRNAs in human body fluids, including blood, urine, and saliva, makes them a potential readily accessible prognostic biomarker for certain diseases [38]. Specifically, a recent study highlighted that three specific piRNAs (piR-009228, piR-016659, and piR-020496) were differentially expressed in the plasma-derived exosomes of pregnant women carrying fetuses with congenital malformations [39].

Long non-coding RNAs (lncRNAs) represent a heterogeneous class of ncRNAs (≥200 nt) that are able to act as positive or negative regulators of gene expression and cellular functions [17]. According to their genome localization, they can be classified into long intervening/intergenic noncoding RNAs, intronic lncRNAs, sense lncRNAs, and antisense lncRNAs [40]. They can influence transcription both in *cis* (nearby genes) and in *trans* (distant genes) by binding to DNA or DNA-bound proteins [17]. In the cytoplasm, they regulate mRNA stability by acting also as “sponges” for the binding of a miRNA with a mRNA [41]. XIST is one of the earliest examples of lncRNA that is both necessary and sufficient for X chromosome inactivation in XX female cells [42]. LncRNAs modulate a wide variety of biological processes, such as cellular differentiation, maintenance of stem cell pluripotency, and development of tissues and organs, contributing to the onset of human diseases [17,43]. They are implicated in different pregnancy-related complications, including preeclampsia [44], GDM [45], intrauterine growth restriction (IUGR) [46], and missed abortions [47].

Circular RNAs (circRNAs) are long non-coding RNAs usually formed by alternative splicing of pre-mRNA, where the 5′ upstream splice acceptor is joined to the 3′ downstream splice donor in a process named “backsplicing” [48]. Growing evidence reveals that circRNAs behave as a competing endogenous RNA (ceRNA) by acting as a miRNA “sponge”, thus reducing miRNA regulatory effect on the expression of the downstream genes [49]. CircRNAs have a significant role in regulating gene expression in gametogenesis, embryogenesis, and cell differentiation [50]. Aberrant expression of circRNAs appears to be related to many diseases, including neurological disorders, diabetes, cardiovascular diseases, and cancer [48], and circRNAs are detectable in the peripheral whole blood and blood cells [51]. In detail, specific circRNAs were found to be downregulated in plasma of pregnant women with GDM [52]. Furthermore, a differential expression profile of circRNAs [53,54], as well as a modified circRNA–miRNA–mRNA network [49], were also associated with PE.

**Figure 2:**
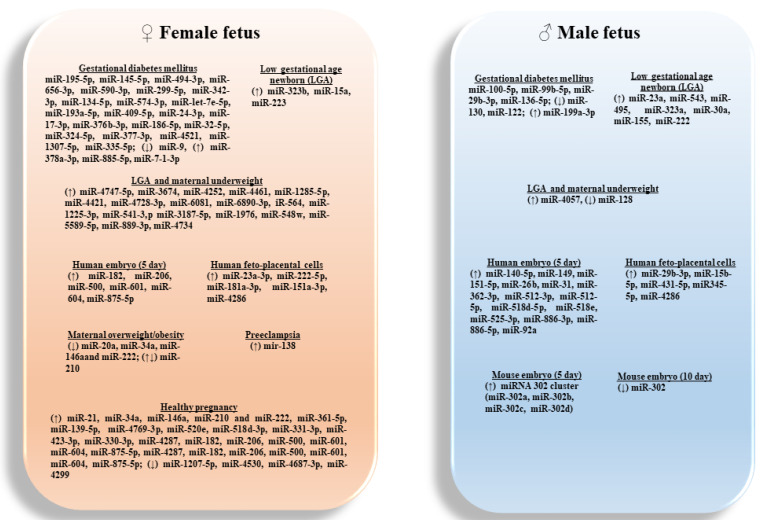
Sex-specific associated of candidate miRNAs expressed in placentas, amniotic fluids and embryos, with pregnancy-related conditions.

Overall, the increasing knowledge about the association between ncRNA profiles and human pregnancy-related conditions suggests that these classes of RNAs might act as feasible and useful biomarkers for several diseases (Figure 2).

## 3. The Importance of Sexual Dimorphism in Placental ncRNAs Expression

The placenta is a temporary and sex-specific endocrine organ that regulates maternal–fetal exchanges, with a central role in fetal growth and development [55]. Sex differences in placenta become evident from the beginning of pregnancy. Specifically, a male fetus grows faster than a female fetus already in the pre-implantation phase, and it invests more in fetal than placental development [56]. Consequently, male placentas are smaller in size and more vulnerable to adverse events during pregnancy than female placentas [56]. These findings indicate that placental adaption to maternal environmental stimuli can influence fetus programming with long-term effects [57]. Of note, the plasticity of the placenta allows fetal signals to influence in a sex-specific manner maternal health as well [4]. However, the complex interaction between placental function and fetal development is not well known. To this end, in the last few years, several studies have tried to shed light on the mechanisms related to sex-specific fetus adaptation during pregnancy by using omics approaches as well [58]. Metabolomics of mouse placentas showed that sexual dimorphism contributes to a different placental adaptation to nutrition, stress, hormones, and chemicals to ensure normal fetal development [58]. A growing amount of data has shown that placental miRNAs and lncRNAs have a regulatory role in fetal developmental programming [59] and pregnancy progression [60,61], as well as in genomic imprinting and susceptibility to diseases later in life [62], by influencing many signaling pathways (Table 1).

A recent microarrays analysis of sex-specific miRNA expression performed in healthy placentas (30 girls and 30 boys; ENVIRONAGE study) showed that 142 miRNAs were significantly associated with the newborn’s sex. Of note, 76 miRNAs had a higher expression and 66 miRNAs had a lower expression in placentas from females compared with the males. Among them, miR-361-5p had the highest expression in girls, and miR-4646-5p had high expression in boys. Placental miRNA-mRNAs interactome analysis showed that a higher expression of miR-361-5p, miR-139-5p, and miR-4769-3p was associated with the downregulation of *PCDH11X*, protocadherin 11 X-linked gene, in girls compared with boys. The *CD99* gene, downregulated in girls, correlated with higher expression of miR-520e, miR-518d-3p, miR-331-3p, miR-423-3p, and miR-330-3p. Furthermore, a higher expression of miR-4287 downregulated *DDX3Y*, DEAD (Asp-Glu-Ala-Asp) box helicase 3 Y-linked in girls, compared with boys. Instead, the downregulation of miR-1207-5p, miR-4530, and miR-4687-3p inversely correlated with *KDM6A*, lysine (K)-specific demethylase 6A, while the downregulation of miR-4299 correlated with CDK16, cyclin- dependent kinase 16, in girls compared with boys. Overall, these data highlighted a sex-specific miRNA involvement in the modulation of metabolic, immunological, apoptotic and neurogenesis processes in healthy pregnancy [63]. As part of the study of the ENVIRONAGE birth cohort, Tsamou and colleagues observed that a higher expression of miR-21, miR-34a, miR-146a, miR-210, and miR-222 associated with longer telomere length in healthy female placentas and not in male ones. Shorter telomeres length is related to increased senescence, and miRNAs have a fundamental role in regulating these processes. These miRNAs have a critical role in inflammation (miR-21, miR-146a), oxidative stress (miR-210), cell cycle (miR-21, miR-34a, miR-210, and miR-222), cellular senescence (miR-21, miR-34a, miR-146a, miR-210), and apoptosis (miR-21), which are key cellular processes in aging and in telomere length regulation. Although other miRNAs might be involved in the aging process, this study indicates that sex differences influence telomere length from early life [64]. As the abovementioned miRNA are also involved in cellular processes underlying obesity, Tsamou and colleagues evaluated their expression in relation to pre-pregnancy body mass index (BMI). Results showed that higher maternal pre-pregnancy BMI was associated with lower placental expression of miR-20a, miR-34a, miR-146a, miR-210, and miR-222 in girls but not in boys. Moreover, gestational weight gain influenced the expression of the above-evaluated miRNAs [65]. On the contrary, as far as miR-210 is concerned, another study showed higher expression of miR-210 in placentas of overweight (OW) and obese (OB) women with female fetuses, compared with the male ones. This finding was associated with an increased expression of the inflammatory molecules TNFα and NFκB1 (p50) and with a reduced expression of the proteins ISCU (iron sulfur cluster assembly enzyme) and NDUFA4 (NADH dehydrogenase subcomplex), which are involved in mitochondrial function [66]. Moreover, the authors showed that the increase in miR-210 expression was prevented by silencing NFκB1 with specific siRNA in TNFα-treated cells. Consequently, they hypothesized that maternal inflammation led to the activation of the transcription factor NFκB1, responsible for the increased expression of miR-210 and subsequent mitochondrial dysfunction in female placentas from OW and OB women [66]. This hypothesis is strengthened by the observation that ISCU and NDUFA4 genes are directly regulated by miR-210 in the first trimester primary extravillous trophoblasts (EVTs) [67]. The finding that women with PE expressed higher placental and circulating levels of miRNA-210 [68] suggested that miR-210 contributes to the pathogenesis of PE by altering mitochondrial function and the first trimester trophoblast function [67].

Sex-dependent miRNA and mRNA expression has been observed in placentas from the extremely low gestational age newborn cohort (ELGAN study; < 28 weeks gestational age). Genome-wide RNA-sequencing analysis showed that 59 miRNAs were differentially expressed between sexes. Of these, miR-23a, miR-543, miR-495, miR-323a, miR-30a, miR-155, and miR-222 were upregulated in male, and miR-323b, miR-15a, and miR-223 were increased in female placentas. All of them are differently involved in placental functions. Specifically, miR-23a, miR-495, miR-222, and miR-223 target genes were associated with PE, and miR-323a with ectopic pregnancy. Interactome analysis revealed that the mRNAs upregulated in female placentas included genes involved in epigenetic control (HDAC1, HDAC8) and cell cycle progression (CDKN1B). A large number of these miRNAs are located on the X-chromosome, suggesting for some of them the escape from XCI (X chromosome inactivation). It is worth noting that miR-543, miR-495, miR-323a, and miR-323b are imprinted miRNA genes belonging to the maternally expressed C14CM miRNA cluster involved in prenatal growth and placentation [69]. Recently, the same groups examined sexually dimorphic mRNA–miRNA expression in relation to pre-pregnancy BMI in placentas from ELGAN study subjects. Data showed that 572 mRNAs associated with pre-pregnancy underweight status in male placentas. Almost all of them (96%) were overexpressed and were involved in known regulatory networks related to nutrition, growth, and angiogenesis, including eukaryotic initiation factor 2 (EIF2) signaling, mammalian target of rapamycin (mTOR) signaling, insulin-like growth factor 1 (IGF-1) signaling, and vascular endothelial growth factor (VEGF) signaling. Of note, only 43 of 572 mRNAs were targets of the two miRNA (miR-4057 and miR-128) that were associated with underweight status in male placentas. In female placentas 18 overexpressed miRNA (miR-4747-5p, miR-3674, miR-4252, miR-4461, miR-1285-5p, miR-4421, miR-4728-3p, miR-6081, miR-6890-3p, iR-564, miR-1225-3p, miR-541-3,p miR-3187-5p, miR-1976, miR-548w, miR-5589-5p, miR-889-3p, miR-4734) were associated with underweight status. The differentially expressed genes in male placentas were also expressed in female placentas. However, none of the mRNAs were associated with underweight status, nor were they a target of the 18 overexpressed miRNAs in female placentas. The authors hypothesized that the altered gene expression in male placentas can represent a protection against intrauterine growth restriction. None of the genes were differentially expressed in overweight and obese status. Of note, the authors confirmed the association between miR-210 and overweight status [70].

Sedlmeier and colleagues revealed a sex-specific response to maternal supplementation with n-3 long-chain polyunsaturated fatty acid (LCPUFA) during pregnancy (INFAT study). They observed that n-3 LCPUFA supplementation had a more evident impact on gene expression in female placentas than in male [71]. The same authors analyzed the effect of n-3 LCPUFA supplementation on placental miRNA expression in a sex-specific manner. They highlighted a differential expression of miR-99a between sexes. MiR-99a can target the expression of *mTOR*, *SLC7A5*, and *SLC6A6* genes, encoding the nutrient sensor *mTOR* and the amino acid transporters LAT1 and TauT, respectively. The authors observed that miR-99a, *mTOR*, and *SLC7A*5 mRNA levels were higher in male placentas compared with female ones, and n-3 LCPUFA supplementation stimulated elevated levels of miR-99a, mTOR, and SLC7A5 mRNA only in female placentas. Amino acids levels were analyzed in maternal plasma (at 15 and 32 weeks of gestation), placenta, and cord plasma. Increased tryptophan (Trp) levels were found in maternal plasma (32 weeks), with significantly higher levels in mothers with female offspring, whereas lower levels of taurine (Tau) were detected in cord plasma of male offspring after supplementation. Significant associations were found for Trp and Tau and for miR-99a mTOR, SLC7A5 mRNA mTOR, and miRNA-99a with offspring body composition. No amino acids changes were detected in placenta after supplementation. The authors concluded that miRNA-99a could be a novel regulator of a complex network in placenta and in maternal and fetal compartments, regulating amino acids homeostasis and offspring’s body composition [72].

Recently, Guo and colleagues through the analysis of miRNAs expression in human placenta evaluated population-associated differences. To this end, they examined miRNA expression in placentas from women belonging to four major human ethnic groups (African Americans, European Americans, South Asians, and East Asians). The authors highlighted that 139 miRNAs were differently expressed among the four populations. Specifically, the expression of 14 miRNAs was higher in male placentas and 18 miRNAs were overexpressed in female ones. Male-associated miRNAs potentially regulated 46 mRNAs and were mostly associated with glutamate receptor signaling and endocrine processes, whereas female-associated miRNAs had 65 mRNAs as potential targets, influencing functions linked to steroid hormones, such as estradiol and glucocorticoid response, as well as cell differentiation and metabolic processes. Results revealed that among the 12 evaluated demographic variables, population identity and the sex of the offspring contributed to miRNA variation [73].

Overall, these data increased awareness of the importance of studying miRNAs and mRNAs interaction in a sex-specific manner in human placenta to better understand sex differences in response to perinatal environment and susceptibility to the diseases later in life (Table 1).

**Table 1:** Sex-specific ncRNAs expression in placentas.

**Model**	**ncRNAs**	**Sex Regulation** **Female ♀ vs. Male ♂ Fetus**	**Targeted Genes/Processes**	**Study**
Overweight and obese pregnant women	miRNAs	♀ (↑) miR-210	♀ (↑) TNFα and NFκB1; mitochondrial dysfunction	[66]
Increasing maternal pre-pregnancy BMI	miRNAs	♀ (↓) miR-20a, miR-34a, miR-146a, miR-210, and miR-222	♀♂ Cell proliferation, cell growth and invasion, inflammation, angiogenesis, and oxidative stress	[65]
Healthy human pregnancies	miRNAs	♀ (↑) miR-21, miR-34a, miR-146a, miR-210, and miR-222	♀♂ Inflammation, oxidative stress, cell cycle, cellular senescence, and apoptosis ♀ longer placental relative telomere length	[64]
Human low gestational age newborns (<28 weeks gestational age)	miRNAs	♀ (↑) 27 miRNAs, including miR-323b, miR-15a, and miR-223 ♂ (↑) 32 miRNAs, including miR-23a, miR-543, miR-495, miR-323a, miR-30a, miR-155, and miR-222	♀♂ Embryo implantation and development ♀ X chromosome inactivation, epigenetic control (HDAC1, HDAC8), and cell cycle progression (CDKN1B)	[69]
Human low gestational age newborns (<28 weeks gestational age)	miRNAs	♀ (↑) miR-4747-5p, miR-3674, miR-4252, miR-4461, miR-1285-5p, miR-4421, miR-4728-3p, miR-6081, miR-6890-3p, iR-564, miR-1225-3p, miR-541-3,p miR-3187-5p, miR-1976, miR-548w, miR-5589-5p, miR-889-3p, miR-4734 ♂ (↑) miR-4057, (↓) miR-128	♀♂ Underweight status ♀ No target genes associated with underweight status ♂ regulate 43 genes related to nutrition, growth, and angiogenesis	[70]
Healthy human pregnancies	miRNAs	♀ 76 (↑), and 66 (↓) miRNAs, most significant miR-361-5p	♀♂ Metabolic, immunological, apoptotic, and neurogenesis processes underlying sex differences	[63]
		♀ (↑) miR-361-5p, miR-139-5p, miR-4769-3p	(↓) *PCDH11X*	
		♀ (↑) miR-520e, miR-518d-3p, miR-331-3p, miR-423-3p, and miR-330-3p	*(↓)* *CD99 gene*	
		♀ (↑) miR-4287	*(↓)* *DDX3Y*	
		♀ (↓) miR-1207-5p, miR-4530, and miR-4687-3p	*(↑)* *KDM6A*	
		♀ (↓) miR-4299	*(↑)* *CDK16*	
Healthy pregnancy from women with/without daily supplement with n-3 LCPUFA	miRNAs	♂ (↑) basal miR-99a	♀♂ Maternal-fetal amino acids homeostasis	[72]
		♀ (↑) miR-99a following supplementation	♂ ↑ basal mTOR and SLC7A5 mRNA ♀ ↑ mTOR and SLC7A5 mRNA following supplementation	
Healthy pregnancies from major ethnic groups: African Americans, European Americans, South Asians, and East Asians	miRNAs	♂ (↑) 14 miRNAs 371a-5p,372-3p,181b-2-3p, let-7g-3p, 185-3p, 3615, and 3158-3p	♂ glutamate receptor signaling and endocrine processes	[73]
		♀ (↑) 18 miRNAs	♀ steroid hormones, estradiol, and glucocorticoid response; differentiation and metabolic processes	

↑ Increases; ↓ decreases; ♀ female; ♂ male; BMI, body mass index; CD99, cluster of differentiation 99; CDK16, cyclin-dependent kinase 16; CDKN1B, cyclin-dependent kinase inhibitor 1B; DDX3Y, DEAD-box helicase 3 Y-linked; HDAC, histone deacetylase; KDM6A, lysine demethylase 6A; miRNA, microRNA; mTOR, mammalian target of rapamycin; ncRNA, non-coding RNA; NFκB, nuclear transcription factor kappa B; PCDH11X, protocadherin 11 X-linked; n-3 LCPUFA, n-3 long-chain polyunsaturated fatty acids; SLC7A5, solute carrier family 7 member 5; TNFα, tumor necrosis factor α.

## 4. Sex-Based ncRNAs Expression in Fetal Development

Embryo and fetus growth and development are tightly regulated processes involving embryonic genome and transcriptional sexual dimorphism as well as epigenetic and maternal factors that control the sequence of stage-specific changes leading to a fully developed organism [13]. In this complex context, an increasing number of ncRNAs appear to regulate gene transcription at every stage of embryo and fetus development [74]. Several studies have shown the importance of miRNAs and piRNAs in human reproduction [13]. Specifically, they are involved in the regulation of pre- and post-fertilization stages, such as sex-specification, commitment, and maintenance of mammalian gonads; embryonic implantation and early development; silencing of X-linked gene expression; and maternal-embryo cross talk [27,74]. The increasing knowledge about stage-specific ncRNA profiles during fetal development is expected to take a step forward in improving assisted reproductive technology (ART) programs as well [74]. Various studies have shown that a differential expression of certain miRNAs was associated with implantation failure [75]. Moreover, an aberrant expression of miRNAs has been observed in reproductive cells/tissues in the context of infertility in humans, both in females [76,77] and males [76], indicating a fundamental role of these ncRNAs in the de-regulation of the reproductive system and early embryogenesis (Table 2).

Indeed, different miRNA profiles seem to reflect embryonic ploidy status (i.e., euploid vs. aneuploid), as well as sexual dimorphism, in human blastocysts (Table 2). It has been shown that miR-140-5p, miR-149, miR-151-5p, miR-26b, miR-31, miR-362-3p, miR-512-3p, miR-512-5p, miR-518d-5p, miR-518e, miR-525-3p, miR-886-3p, miR-886-5p, miR-92a, and RNU48 were highly expressed in cryopreserved human male embryos (day 5 of development) from in vitro fertilization (IVF), and miR-182, miR-206, miR-500, miR-601, miR-604, and miR-875-5p were highly expressed in the female ones. Genes targeted by these miRNAs belong to several pathways that are essential for embryo development, cell cycle, and apoptosis, indicating the importance of ncRNAs in these processes and their possible role as diagnostic biomarkers of infertility and prognostic tools of embryo development [78].

In another complex study, sex-specificity was identified in miRNA expression in mouse embryonic stem cells (ES) at different day (D) of differentiation (i.e., D0, D2, D5). Specifically, the authors highlighted that the miR-302 genomic cluster (i.e., miR-302a, miR-302b, miR-302c, miR-302d) was more expressed in male ES at D5, compared with the female. The authors identified as putative miRNA targets the chromatin remodeling and the E2F-dependent transcription repressors Ari4a and Arid4b, involved in diverse cellular processes including proliferation, differentiation, and cell fate determination. Decreased miR-302 level at D10 in male ES suggested a specific role of this miRNA during a narrow window of male development [79]. By using next-generation miRNA sequencing, sex-dependent miRNA expression patterns were identified in feto-placental endothelial cells (fpEC) obtained from healthy pregnancies. Nine miRNAs were differentially expressed in male and female fpEC; specifically, miR-29b-3p, miR-15b-5p, miR-431-5p, miR345-5p, were overexpressed in male, and miR-23a-3p, miR-222-5p, miR-181a-3p, miR-151a-3p, miR-4286 were overexpressed in female. The analysis of the potential function of these miRNAs indicated their involvement in endothelial barrier function regulation. Specifically, they modulated adherent junctions, extracellular matrix (ECM) receptor interactions, and focal adhesion, eventually suggesting that male cells have increased barrier resistance compared with female ones. The authors hypothesized that the distinct features of endothelial cells already in utero can have a role in sexual dimorphism of endothelial dysfunction and cardiovascular disease in male versus female [80]. However, the advance in high-throughput sequencing technology, together with sexually dimorphic gene expression analysis, represent valuable tools for explaining the basis of sex differences in fetal programming and development and in the susceptibility to diseases.

## 5. ncRNAs in Pregnancy Complications: Role of Fetal Sex

It is becoming increasingly evident that fetal sexual dimorphism differently influences pregnancy outcomes [4,5,6,7] and that ncRNAs actively participate in every step of this (patho)-physiological condition [13,14] (Table 3). Early pregnancy loss (EPL) has been associated with an increased expression of miR-125a-3p, miR-3663-3p, miR-423-5p, and miR-575 in villous tissue, along with upregulation of miR-122 and let-7c and downregulation of miR-135a in maternal plasma. The target genes of these miRNAs were associated with cell migration, proliferation, implantation, adhesion, angiogenesis, and differentiation; thus, they might be involved in EPL pathogenesis [81]. The higher expression of miR-374a-5p and let-7d-5p detected in maternal plasma (at 12^+0^ –14^+6^ weeks gestation) in small-for-gestational-age (SGA) could be predictive of SGA births [29], whereas the downregulation of circulating miR-324-3p appeared to be associated with ectopic pregnancy [30]. Moreover, the analysis of circulating miRNAs 12 weeks postpartum from women with previous GDM revealed that levels of miR-369-3p were higher in women who developed T2D within 10 years [82]. The involvement of this miRNA in the regulation of inflammatory response led the authors to hypothesize that this miRNA, in association with other clinical/biochemical risk factors, could be useful in monitoring T2D progression [82,83]. It is noteworthy that the observation that several postpartum miRNA profiles, specifically observed in cardio-, cerebro-, and vascular diseases, were observed in women experiencing gestational hypertension, preeclampsia, and intrauterine growth restriction. This finding indicates the possible usefulness of miRNA screening to identify women at higher risk of diseases later in life [84].

Gestational diabetes (GDM) can lead to adverse outcomes, both in mother and child, in the short and long term [31]. Although the molecular mechanisms involved in its pathophysiology are not fully elucidated, ncRNAs appear to have an important role in the pathogenic mechanisms of GDM (reviewed by [31]). Indeed, in the last few months, the impressive number of studies reporting an association between GDM and different classes of ncRNAs, including miRNAs [85,86,87]; circRNAs [52,88,89]; and long ncRNAs [90], support the possible role of ncRNAs as an early predictor of the disease. Nevertheless, a few studies have investigated the ncRNA pattern according to the fetal sex (Table 3). In this regard, Strutz and colleagues showed that maternal diabetes differently affects miRNA expression pattern in human placental endothelial cells (fpEC) obtained from male and female placentas. By using next-generation miRNA sequencing, it was observed that miR-195-5p, miR-145-5p, miR-494-3p, miR-656-3p, miR-590-3p, miR-299-5p, miR-342-3p, miR-134-5p, miR-574-3p, miR-let-7e-5p, miR-193a-5p, miR-409-5p, miR-24-3p, miR-17-3p, miR-376b-3p, miR-186-5p, miR-32-5p, miR-324-5p, miR-377-3p, miR-4521, miR-1307-5p, miR-335-5p, were differentially expressed in females and that only miR-100-5p, miR-99b-5p, miR-29b-3p, and miR-136-5p, were differentially expressed in the male in GDM-fpEC. According to the analysis of the possible biological pathways targeted by the female-associated miRNAs, the altered pathways were “proteoglycans in cancer”, “protein processing in endoplasmic reticulum”, and “Hippo signaling pathway”, a signaling cascade that regulates vascular development, proliferation, and apoptosis. Differently, male-associated miRNAs were linked to “ECM receptor interaction”, which deals with endothelial dysfunctions and diabetes complications. Overall, these data suggest that the female miRNA pattern is more vulnerable to the GDM environment than the male one [91]. In another study, the authors measured miRNA expression in amniotic fluid (AF; 2nd trimester) in women with GDM. Results showed that among the 18 miRNAs changed in GDM-AF, a significant increase in miR-378a-3p, miR-885-5p, and miR-7-1-3p was observed in AF samples from female offspring, whereas only miR-199a-3p increased in the male ones. As the detected miRNA target genes were involved in hepatic pathways, the authors analyzed them in primary human fetal hepatocytes (PHFH) obtained from legally aborted second trimester fetuses exposed to maternal obesity. Female PHFH showed increased expression of miR-885-5p, miR-199-3p, miR-503-5p, miR-1268s, and miR-7-1-3p, whereas no miRNA changes were measured in male PHFH. The overexpression of the abovementioned miRNAs reduced the expression of the target genes *ABCA1,* *PAK4,* and *INSR* involved in cholesterol delivery in the liver and placenta, in hepatocyte proliferation, and fetal growth, respectively. The authors concluded that GDM and maternal obesity share a similar intrauterine environment, and miRNAs, in a sex-specific manner, have a critical role in maternal–fetal communication, thus affecting fetal development [92]. Sex-dependent changes in fetal liver lipid content were observed in fetuses from GDM rats. Specifically, Fornes and colleagues, found increased levels of triglycerides and cholesterol in male livers and opposite changes in the female ones. In males, lipid accumulation was associated with increased expression of PPARγ and the lipogenic genes *FAS* and *ACC1*, and with a reduction in miR-130, which targets PPARγ. Interestingly, although increased PPARδ expression was observed in livers of both male and female fetuses of GDM rats, this feature occurred in association with the reduction in different miRNAs that target PPARδ namely, miR-9 in females and miR-122 in males. The authors concluded that sex-specific alterations in fetal liver might result in different adaptive responses later in offspring life [93].

Moreover, since male sex of the fetus appears to be associated with GDM [8], future studies on miRNA expression analysis should include fetal sex as an additional risk factor for maternal metabolic disorders.

Preeclampsia (PE) is a complex syndrome with high morbidity and mortality in both mother and fetus. It is usually diagnosed after week 20 of gestation and is characterized by maternal hypertension and proteinuria [14]. An increasing number of studies have been investigating PE etiopathogenesis, showing that ncRNAs, especially miRNAs [14], circRNAs [94,95,96], and lncRNAs [44] detectable in both maternal placenta and plasma, have a crucial role in endothelial angiogenic function and placentation [97,98,99].

In addition, even though women carrying female fetuses seem to be at higher risk for PE [10], there are very few studies investigating the possible relationship between miRNA expression and fetal sex (Table 3). Li H and colleagues did not find any difference in exosomal miRNA expression between female or male fetuses in plasma of women with PE [100], whereas Leseva’s group observed mir-138 upregulation in placentas of mothers carrying female fetuses. Mir-138 regulates many cellular processes, including cell migration, epithelial-to-mesenchymal transition, cell cycle progression, and cell differentiation, which are connected to placental adaption [32].

However, at present, incomplete knowledge of the molecular mechanisms governing PE, along with tissue-specific miRNA expression and women’s ethnicity [99,100], make it difficult to use miRNAs as possible diagnostic biomarkers.

**Table 3:** Fetal sex-specific ncRNAs expression in pregnancy complications.

**Model/Tissue**	**ncRNAs**	**Sex Regulation** **Female** **♀** **vs. Male** **♂** **Fetus**	**Targeted Genes/Processes**	**Study**
GDM(human feto-placental endothelial cells)	miRNAs	♀ differently expressed: miR-195-5p, miR-145-5p, miR-494-3p, miR-656-3p, miR-590-3p, miR-299-5p, miR-342-3p, miR-134-5p, miR-574-3p, miR-let-7e-5p, miR-193a-5p, miR-409-5p, miR-24-3p, miR-17-3p, miR-376b-3p, miR-186-5p, miR-32-5p, miR-324-5p, miR-377-3p, miR-4521, miR-1307-5p, miR-335-5p	♀ signaling cascade regulating vascular development, proliferation, and apoptosis	[91]
		♂ differently expressed: miR-100-5p, miR-99b-5p, miR-29b-3p, miR-136-5p	♂ ECM receptor interaction: endothelial dysfunctions and diabetic complications	
GDM(fetuses rat)	miRNAs	♂ (↓) miR-130	♂ (↑) PPARγ and the lipogenic genes *FAS* and *ACC1*: liver lipids accumulation	[93]
		♀ (↓) miR-9 and ♂(↓) miR-122	♀♂ (↑) PPARδ liver alterations	
GDM (human amniotic fluid; 2nd trimester)	miRNAs	♀ (↑) miR-378a-3p, miR-885-5p, miR-7-1-3p ♂ (↑) miR-199a-3p	♀♂ (↓) *ABCA1*, *PAK4*, and *INSR* involved in cholesterol delivery in the liver and placenta, in hepatocyte proliferation, and fetal growth	[92]
PE (Plasma)	miRNAs	♀♂ No differences in exosomal miRNAs		[100]
PE (Placenta)	miRNAs	♀ (↑) mir-138	♀♂ Cell migration, cell cycle progression, and cell differentiation: placental adaption	[32]

↑ Increases; ↓ decreases; ♀ female; ♂ male; ABCA1, ATP-binding cassette subfamily A member 1; ACC1, acetyl-coenzyme A carboxylase 1. ECM, extracellular matrix; GDM, gestational diabetes; INSR, insulin receptor; miRNA, microRNA; ncRNA, non-coding RNA; PAK4, p21-activated kinase 4; PE, preeclampsia; PPAR, peroxisome proliferator-activated receptors.

## 6. Conclusions

A greater knowledge of the sex-specific mechanisms governing fetal development and pregnancy is fundamental for properly managing pregnancy-associated complications. In the last years, the awareness that ncRNAs have a crucial role in the regulation of cellular processes encouraged studies aimed at revealing the mechanisms through which this category of RNAs regulates gene expression. Moreover, the expression of ncRNAs in almost all body tissues makes them promising biomarkers for diagnosis, treatment, and follow-up of human diseases.

This review is focused on the presence, expression, and function of ncRNAs in relation to placental function, fetal development, and maternal pregnancy-related diseases, considering possible clinical differences depending on the sex of the fetus. Although an increasing number of studies highlighted that different types of ncRNAs—including miRNAs, piRNAs, lncRNAs, and circRNAs—actively participate in every step of pregnancy progression, data including fetal sex as a central variable are still limited and are mainly related to differential miRNA expression.

This overview of the available literature highlights that the mechanisms of action of ncRNAs in regulating gene expression are very complex. Different categories of ncRNAs can interact with each other (e.g., miRNAs and lncRNAs), thus adding further complexity to the comprehension of genes regulation. NcRNA profiles differed according to the analyzed body tissues, stage of pregnancy, and mother’s health and ethnicity. In this context, fetal sex represents an additional although poorly studied variable that needs to be added to increase the knowledge on ncRNAs.

Of note, in the era of personalized medicine, advances in fetal sex-specific research are needed. Such a goal may be achieved by combining multilevel approaches including (i) high-throughput sequencing technology to identify novel ncRNAs categories, (ii) detection of tissue-specific expression patterns of selected ncRNAs for a potential RNAs target, (iii) cellular and molecular studies to analyze selected ncRNAs and various interaction networks, and (iv) larger cohorts of clinical data.

Current and developing innovative technologies will allow the analysis, the storage, and the integration of these different kinds of data, thus providing useful information in understanding the sex-specific ncRNA role in pregnancy and related diseases.

## Figures and Tables

**Table 2:** Sex-specific ncRNAs expression in fetal development.

**Model/Tissue**	**ncRNAs**	**Sex Regulation** **Female ♀ vs. Male ♂ Fetus**	**Targeted Genes/Processes**	**Study**
Cryopreserved human embryos (day 5 of development)	miRNAs	♀ (↑) miR-182, miR-206, miR-500, miR-601, miR-604, miR-875-5p ♂ (↑) miR-140-5p, miR-149, miR-151-5p, miR-26b, miR-31, miR-362-3p, miR-512-3p, miR-512-5p, miR-518d-5p, miR-518e, miR-525-3p, miR-886-3p, miR-886-5p, miR-92a	♀♂ Embryo development, cell cycle, and apoptosis	[78]
Mouse embryonic stem cells	miRNAs	♂ (↑) miR-302a, miR-302b, miR-302c, miR-302d, at differentiation day 5 ♂ (↓) miR-302, at differentiation day 10	♀♂ Chromatin remodeling; proliferation, differentiation, and cell fate determination	[79]
Human feto-placental endothelial cells from healthy pregnancies	miRNAs	♀ (↑) miR-23a-3p, miR-222-5p, miR-181a-3p, miR-151a-3p, miR-4286	♀♂ Endothelial barrier function: adherent junction, ECM receptor interaction, and focal adhesion;	[80]
		♂ (↑) miR-29b-3p, miR-15b-5p, miR-431-5p, miR345-5p	♂ cells have increased barrier resistance compared with female cells	

↑ Increases; ↓ decreases; ♀ female; ♂ male; ECM, extracellular matrix; miRNA, microRNA; ncRNA, non-coding RNA.

## Data Availability

Not applicable.
